# Blockchain-Based Trusted Data Management with Privacy Preservation for Secure IoT Systems

**DOI:** 10.3390/s25144344

**Published:** 2025-07-11

**Authors:** Haojie Zhou, Hongmin Gao, Zhaofeng Ma, Guanhui Lai

**Affiliations:** 1 School of Cyberspace Security, Beijing University of Posts and Telecommunications, Beijing 100876, China; haojiezhou@bupt.edu.cn (H.Z.); mzf@bupt.edu.cn (Z.M.); 2 Beijing University of Posts and Telecommunications-China Mobile Communications Group Co., Ltd. Joint Institute, Beijing 100876, China; gaohongmin@chinamobile.com; 3 China Mobile Information Technology Co., Ltd., Beijing 102206, China; 4 Beijing Key Laboratory of Trusted Regulation and Governance for Artificial Intelligence, Beijing 102206, China; 5 Dongguan Rail Transit Co., Ltd., Dongguan 523073, China

**Keywords:** blockchain, CKKS, decentralized identity, IoT, multi channel

## Abstract

With the explosive growth of the Internet of Things (IoT), the traditional single data sharing scheme has difficulty satisfying the data sharing needs of both same-domain and cross-domain IoT devices. In order to realize efficient data sharing of IoT devices in the same domain with data privacy protection and efficient collaboration between IoT devices in different domains, this paper proposes a trusted data sharing scheme in IoT systems based on multi-channel blockchain. The scheme adopts a multi-channel mechanism to isolate the ledger data between IoT devices of different domains; IoT devices of the same domain utilize hybrid encryption to achieve efficient data sharing through smart contracts, and IoT devices of different domains utilize the CKKS algorithm to achieve cross-domain data sharing with privacy protection through proxy nodes (PNs). In addition, this paper adopts decentralized identity (DID) to achieve autonomous identity management to avoid privacy leakage in IoT devices and adopts InterPlanetary File System (IPFS) to store data files to solve the blockchain storage capacity limitation problem. The security analysis proves that this scheme satisfies the IND-CPA security model, and the performance analysis proves that this scheme has good utility in trusted data sharing of IoT devices.

## 1. Introduction

The Internet of Things (IoT) is an important component of next-generation information technology and is accelerating the deep integration of the physical world and the digital space. By connecting a large number of heterogeneous sensing devices to the network, the IoT enables real-time sensing and intelligent response to environmental conditions, device behavior, and user needs, and is widely used in industrial manufacturing, smart cities, energy management, and other scenarios. With the rapid growth in the number of IoT devices and the continuous expansion of business needs, IoT systems are becoming highly distributed, heterogeneous, and multi-domain. In this context, achieving secure, efficient, and trustworthy data sharing within and across domains has become a core issue for ensuring the reliable operation of IoT systems and the realization of data value.

In large-scale IoT systems, centralized servers not only face bottlenecks in computation and storage, but also may become targets of network attacks, thus affecting the stability of the whole system. In addition, the access control mechanism is difficult to cope with the complex permission management and dynamic authorization requirements in cross-domain scenarios, limiting security and flexibility during data sharing. Although blockchain technology has been introduced to trusted data sharing in IoT as a result of its decentralized and tamper-resistant features, it is difficult for current research to take into account the differentiated needs of intra-domain and cross-domain scenarios. Specifically, intra-domain scenarios such as the collaboration of multiple IoT devices in smart factories need to support high throughput data sharing and access control, while cross-domain scenarios such as the collaboration of manufacturing and logistics IoT devices require secure computation between IoT devices from different domains without exposing the original data. In addition, existing schemes tend to ignore key issues such as identity privacy leakage, blockchain storage capacity limitation, and the computational overhead of privacy protection mechanisms.

In this context, decentralized identity (DID), CKKS-based homomorphic encryption algorithm, and on-chain off-chain collaborative storage technology provide new ideas for solving the above challenges. DID technology effectively avoids the risk of privacy leakage caused by centralized storage of identity information by empowering IoT devices to independently manage their identities without relying on the centralized organization to store the identity information [1]. The CKKS algorithm, as a fully homomorphic encryption scheme that supports floating-point operations, allows computational tasks to be performed directly in the ciphertext state, which provides a privacy guarantee of data availability and invisibility for cross-domain IoT devices collaboration [2]. For example, IoT devices in the transportation industry use CKKS encryption technology to provide information on freight transportation prices, while industrial IoT uses CKKS encryption technology to provide information on industrial product production and other data. This data is provided to a third-party computing center for transportation cost calculations under encryption, thereby enabling cross-domain IoT device data collaboration while protecting data privacy. The InterPlanetary File System (IPFS) and blockchain’s on-chain off-chain collaboration mechanism provides an efficient solution to break through the blockchain storage bottleneck [3,4]. IoT devices often need to deal with high-precision and sensitive large-scale data files during the sharing process, and direct on-chain storage will lead to blockchain network congestion and high cost. IPFS encrypts the original data files and stores them in the off-chain nodes through decentralized storage and content addressing technology, and only important information is uploaded to the on-chain.

In our paper, a multi-channel blockchain-based trusted data sharing scheme is put forward to address privacy protection, cross-domain collaboration efficiency, and the storage scalability of data sharing in IoT; the main contributions are as follows:

(1) We propose utilizing the multi-channel ledger isolation mechanism of blockchain to achieve trusted data sharing of IoT devices, which enables low-latency processing of intra-domain data sharing and data privacy protection among cross-domain IoT devices by assigning independent channels to IoT devices in different domains [5,6].
(2) We combine symmetric encryption and asymmetric encryption to design hybrid encryption and automate the execution of smart contracts and data access control to improve the efficiency of intra-domain trusted data sharing while ensuring data privacy protection [7,8]. Furthermore, we introduce a CKKS fully homomorphic encryption algorithm, which supports the computation center in performing aggregation, optimization, and other computation tasks directly on the basis of ciphertext data so as to realize data availability and invisibility.
(3) We adopt DID technology, allowing IoT devices to generate DID independently, avoiding the danger of a single point of privacy leakage caused by centralized identity servers. To solve the blockchain storage capacity limitation problem, blockchain and IPFS are combined to realize an on-chain off-chain synergy mechanism, and only important information is uploaded to the chain so as to realize the trusted sharing of big data files.
(4) We analyzed the privacy-preserving capabilities of this scheme and demonstrated that it is IND-CPA secure. In addition, performance analysis confirms the applicability and effectiveness of the scheme.

## 2. Related Works

The combination of DID technology and data sharing can realize autonomy and control of users’ identities, and DID private key information does not rely on central institutions for distribution and storage; thus, it better avoids the leakage of users’ identity information. A DID-based identification and data sharing mechanism was proposed by Lin et al. [9]. The scheme leverages blockchain to store DIDs and data hashes, ensuring identity permanence and data integrity while integrating IPFS to enhance data availability. By adhering to W3C standards, it improves scalability and interoperability, addressing the security risks and point-of-failure of identity management in centralized servers. Although Lin combined DID with data sharing, the combination of DID and data sharing in more complex IoT scenarios was not explored. Blockchain-based development and normalization of DIDs was explored by Fukami et al. [10]. By comparing centralized and decentralized identities, they analyzed the influence of decentralized identity structures on data sharing, highlighting its significance in digital government and data sharing. Fukami’s comparison of DID and centralized identity highlights the importance of DID in data sharing, which provides a good approach to identity privacy protection for cross-domain and intra-domain data sharing in IoT.

CKKS homomorphic encryption is a homomorphic encryption technology that supports floating-point arithmetic, which allows computation directly on the encrypted data, ensuring data privacy while realizing computational functions. By applying CKKS technology to data sharing, participants of data sharing can share their encrypted data without exposing the original data. The utilization of CKKS homomorphic encryption was proposed by Reddi et al. to enable secured sharing of electronic medical records while preserving privacy [11]. Although the CKKS algorithm can be used to achieve secure data sharing with privacy protection, Reddi did not consider using DID to protect the privacy of identity information. A privacy-enhanced federated learning scheme with CKKS homomorphic encryption to secure model parameters was proposed by Qiu et al. [12]. It achieves the same training performance as FedAvg while reducing communication and computation costs compared to the Paillier-based approach. Additionally, its feasibility for deployment on IoT devices is discussed. Rahulamathavan et al. proposed a redesigned speaker verification system backend using CKKS fully homomorphic encryption to process voice features securely [13]. Horvath-Bojan et al. proposed a privacy-preserving, network-based contact tracing system using 5G and geo-localization technologies. The system relies on collaboration between mobile operators and government agencies, allowing encrypted data exchange to detect contacts with infected individuals. Using CKKS fully homomorphic encryption, the system computes infection likelihood while maintaining privacy [14,15]. A secure and effective multiparty computation model with blockchain and privacy computing techniques was proposed by Li et al. to overcome the difficulties of low trustworthiness, privacy issues, and safety concerns in the sharing of financial data [16]. Qiu, Rahulamathavan, Horvath-Bojan, and Li have conducted extensive research on data privacy protection but have not addressed the differing requirements for data sharing efficiency and privacy protection in the two distinct data sharing scenarios of cross-domain and intra-domain sharing.

Blockchain is a distributed and untamperable ledger technology. At present, as blockchain technology matures, more and more scholars are applying blockchain to data sharing scenarios. With decentralization and non-tampering characteristics, the use of blockchain for data sharing can realize the recording and tracing of the data sharing process, and the blockchain’s smart contract technology can realize automated execution for data. A secured and flexible scheme for in-vehicle digital twin network data sharing was proposed by Wang et al. [17]. The scheme employs a signature of knowledge to protect identity privacy, utilizes smart contracts for traceability, and introduces a verification control mechanism to enhance data sharing flexibility. Additionally, digital twins with consistent states can remove sensitive information, ensuring data synchronization and privacy. Although Wang considered dual privacy protection for data and identity, the scheme did not implement autonomous control of identity. A credible blockchain-based data sharing scheme that utilizes blockchain to avoid data tampering and Paillier cryptosystem to ensure data confidentiality was proposed by Zheng et al. [18]. The scheme supports data transactions and protects transaction information with the (p,t)-threshold Paillier cryptosystem. However, Zheng’s scheme did not achieve autonomous control over identity and data sharing across domains. PrivySharing, a framework based on blockchain for privacy-protected and secured IoT data sharing in smart cities, was proposed by Makhdoom et al. [19]. The framework partitions the blockchain network into several channels, with each channel dedicated to a particular data type and governed by access rules enforced through smart contracts. Although Makhdoom utilized blockchain channels to isolate ledger data, the proposal did not explore cross-channel data collaboration with privacy protection. Zhang et al. proposed an artificial intelligence-driven network framework that leverages blockchain to enable mutual trust data sharing among mobile network operators [20]. The system is implemented on the Hyperledger Fabric and utilizes smart contracts for oversight and finely grained access control to ensure secure, trustless sharing of data. However, data sharing under this scheme is limited by the storage bottleneck of blockchain. A framework based on blockchain for securing and transparently sharing continuous personal health data, complemented by cloud storage, was proposed by Zheng et al. [21]. The system enables users to possess, control, and share their own data within a GDPR-compliant manner while incorporating a machine learning-based data quality inspection module. This approach facilitates high-quality data sharing for healthcare research and commercial applications. Combining blockchain with an attribute-based encryption solution for data sharing was proposed by Ma et al. [22]. A framework for a blockchain-based federation for traced and anonymized sharing of vehicle-to-vehicle data that eliminates the reliance on roadside devices was proposed by Cui et al. [23]. By combining 5G and enhanced Proof-of-Commitment consensus algorithms, the system ensures secure, efficient, and tamper-resistant data exchange for the Internet of Vehicles. A framework for decentralized storage and sharing that integrates Ethernet network, IPFS, and attribute-based encryption to enhance data security and access control was proposed by Wang et al. [24]. The framework enables data owners to define access policies and distribute secret keys while leveraging smart contracts for secure keyword search, ensuring integrity and completeness of search results in decentralized storage systems. Although Zheng, Ma, and others have proposed using blockchain to achieve secure data sharing, these studies have not simultaneously achieved dual privacy protection for data and identity. The limited storage capacity of blockchain is also a key issue that constrains the efficiency of data sharing.

The aforementioned researchers have conducted extensive explorations in the field of data sharing and privacy protection, focusing primarily on four key directions:

(1) Adopting DID systems to achieve autonomous identity management and mitigate centralized privacy risks.
(2) Leveraging homomorphic encryption technologies like CKKS to ensure privacy-preserving computations.
(3) Integrating blockchain with distributed storage systems such as IPFS to address scalability challenges.
(4) Designing blockchain-based access control mechanisms through multi-channel architectures and smart contracts.

However, existing solutions suffer from many limitations, including their failure to simultaneously address the different requirements of intra-domain efficiency and cross-domain privacy in IoT; the lack of an integrated approach to identity autonomy, storage scalability, and fine access control; and the failure to adequately address the tension between decentralized traceability and computationally intensive privacy preservation. To overcome these difficulties, we propose a trustworthy data sharing scheme for IoT devices with privacy preservation based on multi-channel blockchain:

(1) Domain isolation through a multi-channel architecture that enables efficient processing of intra-domain and cross-domain sharing.
(2) A dual-mode security mechanism combining hybrid encryption for efficient intra-domain sharing with CKKS-based privacy computing for cross-domain collaboration.
(3) A synergistic integration of DID-based identity management and IPFS-augmented storage that eliminates centralized vulnerabilities while ensuring system scalability.

## 3. Preliminaries

### 3.1. Decisional Ring Learning with Errors (DRLWE) Assumption

Let the polynomial ring $R=Z\left[X\right]/\left(X^{n}+1\right)$, let *q* be a modulus, let χ be an error distribution, and let $s\in R_{q}$ be a fixed secret. The following are instances where the distinction is needed:

RLWE instance $A_{s,\chi }$: A sample pair (a,b) drawn from the RLWE distribution satisfies

$$a\leftarrow R_{q},\;e\leftarrow \chi ,\;b=as+e\;\mathrm{mod}\;q.$$

Random instance U: A random pair (a,b) drawn from the uniform distribution satisfies

$$a\leftarrow R_{q},\;b\leftarrow R_{q}.$$

The advantage for any probabilistic polynomial time (PPT) distinguisher D, D is that

$$\left|\mathrm{Pr}\left[D^{A_{s,\chi }}\left(1^{\lambda }\right)=1\right]-\mathrm{Pr}\left[D^{U}\left(1^{\lambda }\right)=1\right]\right|$$

is negligible, which means that no polynomial-time algorithm can distinguish between RLWE samples and random samples with non-negligible advantage.

### 3.2. Decentralized Identifier (DID)

DID is an identity identifier that enables verifiable and autonomous identity management in decentralized systems [25]. Unlike traditional identifiers, such as email addresses or usernames, DIDs are fully under the control of the DID subject, independent of any centralized authority. The main goal of DIDs is the provision of a method for identifying entities (people, organizations, devices, etc.) in a manner that ensures privacy, security, and trust without relying on a central registry [26]. The core components of a DID system include the following:

(1) DID Document: A DID document contains metadata about the DID subject, such as public keys for authentication, services offered by the subject, and other relevant information. It serves as a verifiable claim about the identity of the DID subject.
(2) DID Method: The DID method specifies how DIDs are created, updated, and resolved in a decentralized network. For instance, a blockchain-based DID method utilizes smart contracts to manage DID documents securely and immutably.

### 3.3. CKKS Homomorphic Encryption Scheme

CKKS is a homomorphic encryption scheme designed specifically to handle floating-point computations, and is suitable for privacy-preserving computing, machine learning, cloud computing, and other scenarios. It supports approximation algorithms, i.e., it is possible to perform operations of addition and multiplication under encryption while keeping the privacy of the data, so that the computation results are still approximate to those of the original data after decryption [2]. CKKS homomorphic encryption scheme includes the following algorithms:

KeyGen$\left(1^{\lambda }\right)$: An algorithm for key generation takes as input the security parameter λ and outputs a private key sk, a public key pk and an evaluation key evk. It is used to initialize the encryption system and ensure the security of subsequent operations.

Encode(z;Δ): The encoding algorithm takes a complex vector *z* and a scaling factor Δ as input and encodes them into a plaintext polynomial *m*. It converts floating-point numbers into polynomials using an inverse FFT and multiplies by Δ to preserve precision.

Decode(m;Δ): The decoding algorithm uses a plaintext polynomial *m* and a scaling factor Δ as input, then decodes them into a complex vector *z*. It converts the polynomial back to floating-point numbers using FFT and divides by Δ to restore the original values.

Enc*_pk_*(m): The encryption algorithm uses a plaintext polynomial *m* and pk as input and generates *c*. It encrypts the plaintext using the public key and random noise to protect data privacy.

Dec*_sk_*(c): The decryption algorithm accepts sk and *c* as inputs, and outputs the corresponding plaintext polynomial *m* after decryption.

Add$\left(c_{1},c_{2}\right)$: The addition operation takes two ciphertexts $c_{1}$ and $c_{2}$ as input and outputs their homomorphic addition result $c_{\mathrm{add}}$. It performs addition directly on ciphertexts in the encrypted domain.

${\mathrm{Mult}}_{evk}\left(c_{1},c_{2}\right)$: The multiplication operation takes two ciphertexts $c_{1}$ and $c_{2}$ as input and uses the evaluation key evk to compute the homomorphic multiplication result $c_{\mathrm{mult}}$. After multiplication, rescaling is required to control noise and scaling factor growth.

${\mathrm{RS}}_{\ell \to \ell^{\prime }}\left(c\right)$: The rescaling operation takes a ciphertext *c* as input and reduces its scale level *ℓ* to control noise growth, ensuring precision in subsequent computations.

## 4. Overview

### 4.1. System Model

**Figure 1:**
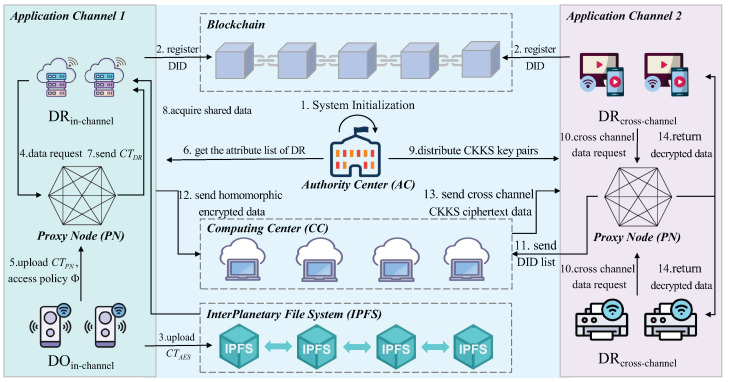
Scheme model for data sharing based on multi-channel blockchain.

The IoT device data sharing system based on multi-channel blockchain includes authority center, IoT device, computing center, proxy node, and IPFS. This system model will be divided into trusted sharing of data between IoT devices within the blockchain channel and trusted privacy computing between IoT devices across the channel. There is a system model as shown in Figure 1.

Authority Center (AC): the AC is responsible for managing the IoT devices. Any devices that join the IoT system need to be authenticated through the AC. After the authentication, the AC registers the DID for each IoT devices joining the IoT system and adds the IoT devices to a specific blockchain channel.

Internet of Things device (IoT device): the IoT device is the main body for data sharing, using the blockchain multi-channel mechanism to achieve trusted data sharing of IoT devices. Devices within the same channel achieve secure data sharing through a proxy node, while devices across different channels perform privacy computation via the computing center.

Computing Center (CC): The CC is the key infrastructure for enabling secure, privacy-protected collaborative computing between IoT devices across channels within the system. It is specifically designed to process joint computing requests initiated by IoT devices from different blockchain channels. By utilizing the CKKS homomorphic encryption algorithm, all parties’ original sensitive data remains encrypted throughout the entire computing process, thereby achieving “data availability without visibility”.

The CC achieves privacy computing between the cross-channel IoT devices, and the computing center uses homomorphic encryption algorithms to make the data securely computed while maintaining confidentiality to ensure that vulnerable information is kept free from unauthorized access during the computation process.

Proxy Node (PN): The PN is responsible for storing the access control strategy on the blockchain through which data sharing between different IoT devices in the channel is realized by the PN, thus enabling access control at a fine-grained level. In addition, cross-channel data sharing also requires the PN to perform CKKS encryption and decryption calculations to enable secure cross-channel data sharing.

InterPlanetary File System (IPFS): A decentralized distributed storage system, IPFS is used to solve the blockchain storage capacity limitation problem. Each shared file in IPFS has a unique hash value based on its content as an identifier. This hash value enables the file to be precisely located and retrieved within the IPFS network. Files are stored in blocks across multiple nodes within the IPFS network. Since data is distributed across multiple nodes, other nodes can still provide the data in cases where nodes go offline or experience failures. Additionally, incentive mechanisms are used to encourage more nodes to offer distributed storage services, ensuring the long-term availability of data and preventing data loss due to single points of failure.

### 4.2. Threat Model

The threat model of this system assumes that the AC is honest, but there may be unauthorized malicious entities that want to obtain shared data information from it or maliciously enter wrong data to corrupt the system computation results. Potential malicious entities include outside, compromised PN, compromised CC, and colluding PN + CC. We identified the adversary’s threat model as known ciphertext model, which is a threat model in which unauthorized malicious entities want to obtain information from the ciphertext to break the confidentiality of the encrypted information. The capabilities of potential adversaries in the threat model are shown in Table 1.

### 4.3. Security Requirement

In this paper, the IND-CPA security model is adopted to measure the security of encryption schemes with known public key and optional plaintext queries. In the IND-CPA security model, the adversary *A* is free to choose the plaintext and obtain the corresponding ciphertext but cannot distinguish the result of encrypting with two equivalent-length plaintexts. Specifically, the adversary *A* submits two plaintexts $m_{0}$ and $m_{1}$ as a challenge, the system chooses *b* randomly among {0, 1} and returns the ciphertext of $m_{b}$, and *A* tries to guess *b*. The encryption scheme is considered secured if *A* has a negligible advantage.

Initialization: Construct challenger *C* and adversary *A* and initialize the system model.

Setup: The challenger *C* randomly samples s←HWT(h), $e\leftarrow DG(\sigma^{2})$ and $a\leftarrow R_{q_{L}}$, generates the sk=(1,s) and the pk=(b=−as+e,a), and then sends this pk to the adversary *A*.

Query Phase 1: *A* sends query requests to challenger *C*. *A* can freely choose plaintext messages and obtain their corresponding ciphertexts.

Challenge: *A* sends two plaintexts of equivalent length, $m_{0}$ and $m_{1}$, to the challenger *C*. The challenger *C* chooses *b* at random among {0, 1} and returns the challenge ciphertext $CT_{b}$ to *A*.

Query Phase 2: In the same way as in Phase 1, the adversary *A* runs the query.

Guess: The adversary *A* guesses $b^{\prime }\in \left\{0,1\right\}$ based on the ciphertext information.

In the IND-CPA game, the advantage of the adversary *A* is given as follows:

$$Adv_{A}^{IND-CPA}=\left|Pr\left[b^{\prime }=b\right]-\frac{1}{2}\right|.$$

### 4.4. Multi-Channel Blockchain Architecture

Our scheme adopts a multi-channel blockchain architecture, which aims to address the key limitations of single-channel blockchain architecture systems in cross-domain collaboration, especially in large-scale IoT device data sharing scenarios. As shown in Table 2, single-channel architectures have inherent bottlenecks, including unpredictable delays caused by single-channel global transaction ordering, limited scalability due to a single consensus mechanism, and inefficient resource utilization due to full ledger replication.

## 5. Detail of Our Proposed Scheme

In a multi-channel blockchain-based IoT system, the combination of blockchain technology and DID ensures secured and trusted data sharing between devices. The system leverages the decentralized nature of blockchain to store and verify data, while DID serve as a unique and tamper-proof identity for each IoT device. This setup guarantees that data exchanges between devices are secure and protected from external interference.

In the context of IoT, blockchain multi-channel architecture allows different categories of IoT devices to be assigned to distinct blockchain channels, each dedicated to a specific type of data or operation. This separation ensures that data privacy and integrity are maintained, while cross-channel data sharing are securely facilitated through well-defined protocols.

By using DID, each device within the IoT has a verifiable identity, and its data transactions are recorded immutably on the blockchain, enhancing both the trustworthiness and accountability of the system. This model enables efficient and secure data sharing across various devices, ensuring that all sharing remain tamper-resistant and verifiable. The scheme model is shown in Figure 1.

The relevant symbol descriptions in this paper are shown in Table 3. The algorithmic scheme for trusted data sharing between IoT devices based on multi-channel blockchain includes the following stages:

### 5.1. System Initialization

This section describes the system initialization process, including four key steps. First, the system generates the necessary homomorphic encryption parameters, defines the polynomial ring *R* and the modulo chain *Q* to support CKKS homomorphic encryption computation, and stores them publicly on the blockchain to ensure transparency. Next, the IoT device generates DID key pairs via the Elliptic Curve Digital Signature Algorithm (ECDSA) [27] and ensures that the private key is kept only by itself to avoid identity disclosure. Subsequently, the IoT device submits a registration request containing identity attributes and its signature to the authority center, which verifies the validity of the signature, generates a unique DID for it and stores it in the blockchain to achieve verifiable decentralized identity management. Finally, to guarantee the security of trusted computing channels, the system generates CKKS homomorphic encryption keys for each channel, including public-private key pairs and evaluation keys, to support subsequent cryptographic computation.

This initialization process ensures the security of the IoT device’s identity, the feasibility of data encryption computation, and the transparency and verifiability of the overall scheme.

Step 1: Generate system parameters

Setup()→params: Define the polynomial ring as $R=Z\left[X\right]/\left(X^{n}+1\right)$, where *n* is a power of two to ensure efficient transform operations. Choose a precision scaling factor Δ to control the trade-off between precision and noise growth in encrypted computations. Select an integer base p>0 and an initial modulus $q_{0}$, which together define a modulus chain $Q=q_{0}\cdot q_{1}\cdot \cdots \cdot q_{L}$ where each modulus level is given by $q_{\ell }=p^{\ell }\cdot q_{0},\;\mathrm{for}\;0<\ell \leq L.$

This modulus chain allows ciphertext modulus reduction, which is a key feature in CKKS to maintain numerical stability and control noise growth during homomorphic operations. The system parameters, including *n*, Δ, *p*, $q_{0}$, and the modulus chain *Q*, are made public and storing it on the blockchain to assure transparency and reproducibility in encrypted computations.

Step 2: Generate key pairs for DID

$DIDKeyGen\left(\right)\to \left(pk_{DID},sk_{DID}\right)$: The DID registration process is shown in Algorithm 1. IoT devices can autonomously generate DID key pairs. After generating the DID key pair, each IoT device needs to register the DID on the blockchain.

The IoT device generates DID key pairs by selecting Elliptic Curve Digital Signature Algorithm (ECDSA) for identity authentication and recognition [27]. ECDSA was chosen because it balances security and efficiency and is suitable for resource-constrained environments typical of IoT devices.

Select elliptic curve parameters ECDSA.Params=(G,p,h,a,b,n), where *p* is a prime number in a finite field, a,b is the coefficients of the elliptic curve equation, *h* is cofactor, *n* is prime order of *G* and *G* is base point. The DID key generation process is shown in Equation (1). The private key $sk_{DID}$ is randomly selected from the set {1,2,…,n−1}, ensuring that it is a valid scalar for point multiplication on the elliptic curve. The corresponding public key $pk_{DID}$ is then calculated as $sk_{DID}\cdot G$, which is the result of scalar multiplication of the base point *G* by the private key $sk_{DID}$. The calculation process is as follows:

(1) $$\begin{array}{cc} sk_{\mathrm{DID}} & \overset{random}{\leftarrow }\left\{1,2,\ldots ,n-1\right\},\\ pk_{\mathrm{DID}} & =\left(x_{pk},y_{pk}\right)=sk_{\mathrm{DID}}\cdot G. \end{array}$$

The private key is generated and stored by the IoT device itself, and is not stored by the AC. The AC is responsible for verifying the legitimacy of the identity information of the IoT device. The autonomous and controllable DID identity can avoid the leakage of private identity information.
**Algorithm 1** IoT device DID Identity Registration**Input:** IoT devices with attribute set $attribute_{i}$ **Output:** Registered and verifiable $DID_{i}$
  1:**Step 1: Generate Key Pairs**  2:$\left(pk_{DID_{i}},sk_{DID_{i}}\right)\leftarrow {\mathrm{ECDSA}.\mathrm{GenKeyPair}}_{i}\left(\right)$  3:**Step 2: Send Registration Request**  4:IoT device prepares registration request:  5:$Request_{i}=\left(attribute_{i},pk_{DID_{i}},Signature_{i}\right)$  6:IoT device sends $Request_{i}$ to AC.  7:**Step 3: Identity Verification**  8:AC extracts information from $Request_{i}$.  9:AC verifies the signature:10:$\mathrm{Verify}(pk_{DID_{i}},Signature_{i})\to \mathrm{Valid}\;\mathrm{or}\;\mathrm{Invalid}$11:**if** Verification is invalid **then**12:   Reject registration request and terminate.13:**end if**14:**Step 4: Generate DID Document and Attribute List**15:Generate unique DID for the IoT device:16:$DID_{i}\leftarrow \mathrm{GenerateDID}\left(pk_{DID_{i}}\right)$17:Construct DID Document and Store $\left(DID_{i},attribute_{i}\right)$ into the attribute list.18:**Step 5: Register DID on Blockchain**19:Call blockchain registration function:20:Blockchain_Register$\left(DID_{i},Document_{i}\right)$21:**if** Registration successful **then**22:   Return confirmation and $DID_{i}$ to IoT device.23:**else**24:   Return registration failure and terminate.25:**end if**


Step 3: IoT device authentication

$IdentityAuthentication(attribute,Sign_{sk_{DID}}\left(attribute\right))$: The AC checks the attribute information attribute of the IoT device and verifies the correctness of the attribute source by verifying the signature $Sign_{sk_{DID}}\left(attribute\right)$ with the DID public key $pk_{DID}$. The DID signature process is shown in Equation (2), and the DID signature verification process is shown in Equation (3).

During the signing process, the attribute information is first hashed to obtain a fixed-length digest. A random scalar *k* is then chosen, and the corresponding elliptic curve point is computed to derive the signature component *r*. The final signature component $s_{1}$ is computed using the private key $sk_{\mathrm{DID}}$ and the hashed attribute. The signing process is as follows:

(2) $$\begin{array}{cc} h & =\mathrm{HASH}(attribute),\\ k & \overset{random}{\leftarrow }\left\{1,2,\ldots ,n-1\right\},\\ r & ={\left(k\cdot G\right)}_{x}\;\mathrm{mod}\;n,\\ s_{1} & =k^{-1}\left(e+r\cdot sk_{\mathrm{DID}}\right)\;\mathrm{mod}\;n. \end{array}$$

For verification, the recipient checks the validity of the signature by ensuring that both *r* and *s* fall within the acceptable range. The verifier then computes an intermediate value *w* and uses it to derive two scalars $u_{1}$ and $u_{2}$, which are used to reconstruct a point on the elliptic curve. If the computed x-coordinate matches *r*, the signature is deemed valid, confirming the authenticity of the attribute information. The signature verification process is as follows:

(3) $$\begin{array}{cc} \mathrm{If}\;r,s & \notin [1,n-1]\Rightarrow \mathrm{Invalid},\\ w & =s^{-1}\;\mathrm{mod}\;n,\\ u_{1} & =e\cdot w\;\mathrm{mod}\;n,\\ u_{2} & =r\cdot w\;\mathrm{mod}\;n,\\ \left(x_{1},y_{1}\right) & =u_{1}\cdot G+u_{2}\cdot pk_{\mathrm{DID}},\\ \mathrm{If}\;r & \equiv x_{1}\;\mathrm{mod}\;n\Rightarrow \mathrm{Valid}. \end{array}$$

### 5.2. In-Channel Data Sharing

**Figure 2:**
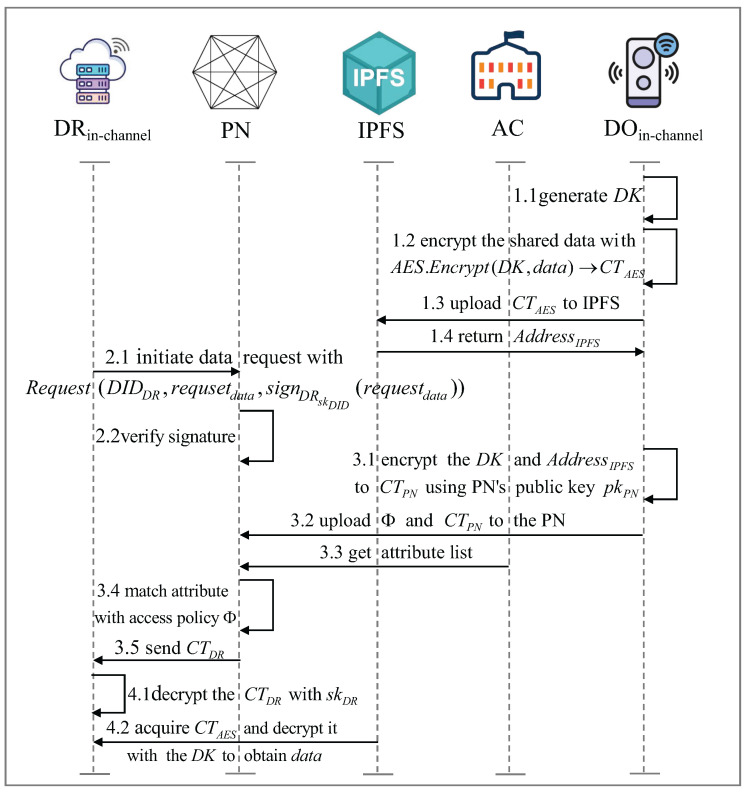
In-channel data sharing.

This process describes secure data sharing between different IoT devices within a blockchain channel. Data sharing within the channel is achieved through hybrid encryption, with symmetric encryption using AES and asymmetric public key encryption using RSA [28]. The entities involved include the data owner (DO) and the data requester (DR). The process consists of four main steps: data encryption and upload, data request, access control, and data acquisition. Figure 2 shows the data sharing process between IoT devices within the channel.

Step 1: Data encryption and upload

The DO encrypts the data using AES encryption in Cipher Block Chaining (CBC) mode:

IV←Random(128)

where IV is a randomly generated 128-bit initialization vector.

The AES encryption process follows:

(4) $$CT_{AES}\left[i\right]=\left\{\begin{array}{cc} Enc(DK,data[0]⊕IV), & i=0\\ Enc(DK,data\left[i\right]⊕CT_{AES}\left[i-1\right]), & i\geq 1 \end{array}\right.$$

where DK is symmetric encryption key, ⊕ denotes bitwise XOR operation, and $CT_{AES}\left[i\right]$ represents the ciphertext block for the *i*-th plaintext block data[i].

The DO then uploads $CT_{AES}$ to IPFS and receives the corresponding storage address:

$$Address_{IPFS}\leftarrow IPFS.Upload\left(CT_{AES}\right)$$

Step 2: Data request

DR submits data request $Request_{data}=\left(DID_{DR},request_{data},sign_{DR_{sk_{DID}}}\left(request_{data}\right)\right)$ to the blockchain PN with a digital signature for verification, where $sign_{DR_{sk_{DID}}}\left(request_{data}\right)$ is a digital signature generated with DR’s $sk_{DID}$.

The PN verifies the request with $Verify(DR_{pk_{DID}},sign_{DR_{sk_{DID}}}\left(request_{data}\right),request_{data})$. Only if verification succeeds, the request proceeds.

Step 3: Access control

The DO encrypts the symmetric key DK and the storage address using the PN’s public key:

(5) $$CT_{PN}=Encrypt\left(pk_{PN},\left(DK,Address_{IPFS}\right)\right)$$

where $pk_{PN}$ is the public key of the PN.

DO formulates an access control policy Φ and submits it along with $CT_{PN}$ to the PN.

The PN retrieves DR’s attribute list from the authority center and evaluates the access control policy:

(6) $$\Phi \left(DR\right)=\left\{\begin{array}{cc} 1, & \mathrm{if}\;\mathrm{DR}\;\mathrm{satisfies}\;\mathrm{policy}\;\Phi \\ 0, & \mathrm{otherwise} \end{array}\right.$$

If the policy is satisfied (Φ(DR)=1), the PN decrypts $CT_{PN}$ and re-encrypts it for the DR using $pk_{DR}$:

(7) $$CT_{DR}=Encrypt\left(pk_{DR},\left(DK,Address_{IPFS}\right)\right)$$

The encrypted information is then sent to the DR.

Step 4: Data acquisition

The DR decrypts $CT_{DR}$ using its private key:

(8) $$DK||Address_{IPFS}=Decrypt\left(sk_{DR},CT_{DR}\right)$$

The DR retrieves the encrypted data from IPFS:

$$CT_{AES}\leftarrow IPFS.Retrieve\left(Address_{IPFS}\right)$$

The AES decryption in CBC mode follows:

(9) $$data\left[i\right]=\left\{\begin{array}{cc} Dec(DK,CT_{AES}\left[0\right])⊕IV, & i=0\\ Dec\left(DK,CT_{AES}\left[i\right]\right)⊕CT_{AES}\left[i-1\right], & i\geq 1 \end{array}\right.$$

Finally, the DR obtains the shared data data. Thus, DR can securely access sharing data as well as keep the integrity and confidentiality of the process intact.

### 5.3. Data Privacy Computation Between Cross-Channel IoT Devices

**Figure 3:**
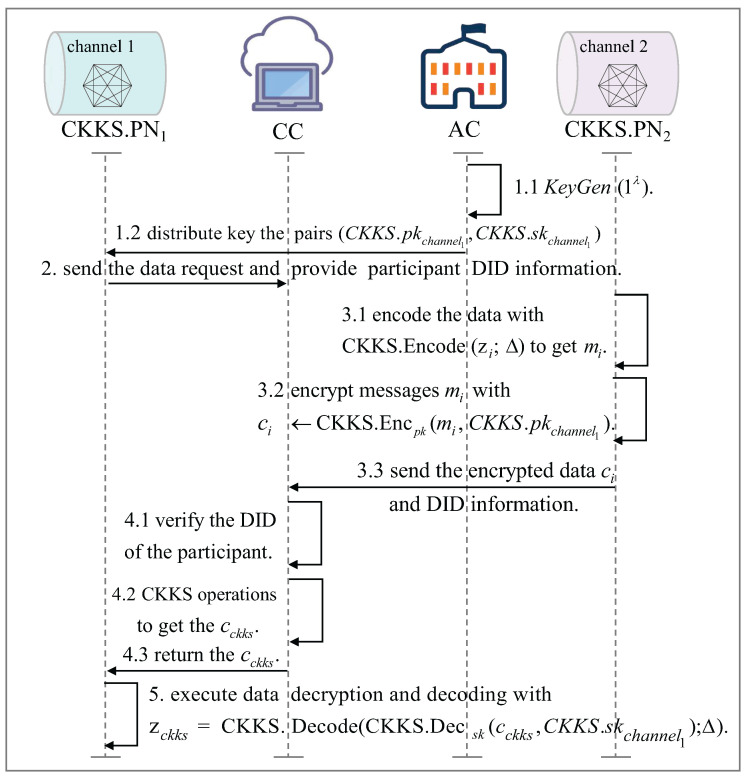
Cross-channel privacy computing.

Figure 3 shows the process of privacy calculation for different channels. In cross-channel IoT device data sharing, we adopt the fully homomorphic encryption scheme CKKS to achieve privacy protected cross-channel data sharing [29,30]. ${\mathrm{CKKS}.\mathrm{PN}}_{1}$ is the CKKS PN of channel 1, and ${\mathrm{CKKS}.\mathrm{PN}}_{2}$ is the CKKS PN of channel 2. Assuming that ${\mathrm{CKKS}.\mathrm{PN}}_{1}$ needs ${\mathrm{CKKS}.\mathrm{PN}}_{2}$’s data to complete some business, ${\mathrm{CKKS}.\mathrm{PN}}_{1}$ and ${\mathrm{CKKS}.\mathrm{PN}}_{2}$ can perform privacy calculations through CC.

CKKS supports floating-point data, making it more suitable for IoT scenarios. Specific application scenarios include hospitals and insurance companies using CKKS to predict or calculate patient premiums while protecting sensitive raw data. Insurance companies request patient medical data from hospitals via cross-domain data requests. Hospitals encrypt medical data (e.g., medical expenses) using the insurance company’s CKKS public key. Insurance companies can encrypt premium calculation model data (e.g., encrypted weighting data) and use CC to calculate patient premiums. Finally, insurance companies decrypt the calculation results.

The specific cross-channel IoT devices trusted data privacy calculation process is as follows:

Step 1: Generate and distribute CKKS keys

CKKS.KeyGen$\left(1^{\lambda }\right)$. For a given security parameter λ, the algorithm initializes multiple parameters. Select modulus *M* as a power-of-two integer satisfying security requirements, define Hamming weight parameter *h* to control private key sparsity, set a large integer *P* as the extended modulus for relinearization, and determine Gaussian noise standard deviation σ to ensure scheme security.

Core sampling operations consist of sample the *s* from the Hamming weight distribution HWT(h), uniformly sample random polynomial *a* from the ring $R_{q_{L}}$, and generate noise polynomial *e* via discrete Gaussian distribution $DG(\sigma^{2})$. Set channel 1’s $sk_{channel1}$ as

$$sk_{channel1}=\left(1,s\right)$$

and channel 1’s $pk_{channel1}$ as

$$pk_{channel1}=\left(b=-as+e,a\right).$$

In the process of generating the evaluation key for our scheme, we begin by sampling a random polynomial $a^{\prime }$ from the ring $R_{P\cdot q_{L}}$, denoted as $a^{\prime }\leftarrow R_{P\cdot q_{L}}$. Simultaneously, an error term $e^{\prime }$ is sampled from a discrete Gaussian distribution with variance $\sigma^{2}$, i.e., $e^{\prime }\leftarrow DG\left(\sigma^{2}\right)$. The channel 1’s evaluation key is then formed as a pair $\left(b^{\prime },a^{\prime }\right)$, where the component $b^{\prime }$ is computed as follows:

$$b^{\prime }\leftarrow -a^{\prime }s+e^{\prime }+Ps^{2}\;\left(\mathrm{mod}\;P\cdot q_{L}\right)$$

In this equation, the term $-a^{\prime }s$ reflects the standard encryption structure, $e^{\prime }$ adds necessary noise for security, and the additional term $Ps^{2}$ ensures correctness during homomorphic multiplication.

As a result, the channel 1’s evaluation key is given by the following:

$$evk_{channel1}\leftarrow \left(b^{\prime },a^{\prime }\right)\in R_{P\cdot q_{L}}^{2}$$

This evaluation key $evk_{channel1}$ enables homomorphic multiplication operations and ensures that decryption remains correct while maintaining both key security and manageable noise growth.

Step 2: Channel 1 sends homomorphic encryption request

Assuming that the IoT device in channel 1 needs some data from channel 2 to complete certain business, IoT device needs to initiate a cross-channel data request to the ${\mathrm{CKKS}.\mathrm{PN}}_{1}$, and then the ${\mathrm{CKKS}.\mathrm{PN}}_{1}$ will initiate a data request to CC and provide DID information of all parties involved in the cross-channel data sharing process. This DID information is used to verify the authenticity of the data provider’s identity.

Step 3: Encode the original data and encrypt it

Both parties involved in privacy computing need to use the CKKS public key of the channel where the requester is located to encode and encrypt the data. We first define the following mappings:

The definition of canonical embedding mapping σ is shown in Equation (12).

(10) $$\begin{array}{cc} \; & \forall m\in C\left[X\right]/\left(X^{N}+1\right),\\ \sigma (m)= & \left(m\left(\xi \right),m\left(\xi^{3}\right),\ldots ,m\left(\xi^{2N-1}\right)\right)\in C^{N} \end{array}$$

where $\xi^{2i-1}$ represents the N primitive roots of the polynomial $X^{N}+1$.

The definition of H as the subring of $C^{N}$ is shown in Equation (11).

(11) $$H=\left\{{\left(z_{j}\right)}_{j\in Z_{M}^{*}}:z_{j}=\bar{z_{-j}}\right\}$$

The definition of natural projection mapping π is shown in Equation (12).

(12) $$\forall t\in H,\pi \left(t\right)=\left(t_{0},t_{1}\ldots t_{N/2}\right)\in C^{N/2}$$

The encoding function is as follows:

$$\begin{array}{cc} C^{\phi (M)/2} & \overset{\pi^{-1}}{\to }H\overset{{\left\lfloor \cdot \right\rceil }_{\sigma (R)}}{\to }\sigma \left(R\right)\overset{\sigma^{-1}}{\to }R\\ z={\left(z_{i}\right)}_{i\in T} & ⟼\pi^{-1}\left(z\right)\\ \; & ⟼{\left\lfloor \pi^{-1}\left(z\right)\right\rceil }_{\sigma (R)}\\ \; & ⟼\sigma^{-1}\left({\left\lfloor \pi^{-1}\left(z\right)\right\rceil }_{\sigma (R)}\right) \end{array}$$

This mathematical transformation describes a sequence of mappings from a complex vector space $C^{\phi (M)/2}$ through intermediate algebraic structures to a ring R.

1. The first mapping $\pi^{-1}$ transforms from $C^{\phi (M)/2}$ into the space H.

2. The next step involves rounding, denoted by ${\left\lfloor \cdot \right\rceil }_{\sigma (R)}$, which projects elements from H into σ(R).

3. The final transformation $\sigma^{-1}$ maps the result back into the ring R.

The notation $z={\left(z_{i}\right)}_{i\in T}$ represents an input vector, which undergoes this series of transformations, ensuring that the final result is within the target ring R.

The encoding process is as follows:

CKKS.Encode(z;Δ). The encoding phase transforms a (N/2)-dimensional complex vector $z={\left(z_{j}\right)}_{j\in T}\subseteq Z{\left[i\right]}^{N/2}$ into a ring element compatible with homomorphic operations. Initially, the map $\pi^{-1}$ projects *z* into the space H. A precision-preserving scaling operation is then applied by multiplying the projected result with a scaling factor Δ, which amplifies the fractional components of *z* to minimize information loss during discretization. Subsequently, a coordinate-wise rounding function ${\left\lfloor \cdot \right\rceil }_{\sigma (R)}$ rounds the scaled value, where σ(R) denotes the ring’s canonical coefficient embedding space.

The encoding process can be expressed as

$$m\left(X\right)=\lfloor \sigma^{-1}\left(\Delta \cdot \pi^{-1}\left(z\right)\right)\rceil \in R$$

The encryption process is as follows:

${\mathrm{CKKS.Enc}}_{pk}\left(m\right)$. Let v←ZO(0.5) and $e_{0},e_{1}\leftarrow DG\left(\sigma^{2}\right)$. Then, we generate the following ciphertext:

(13) $$c=v\cdot pk_{channel1}+\left(m+e_{0},e_{1}\right)\;\left(\mathrm{mod}\;q_{L}\right).$$

Step 4: Perform homomorphic encryption computation

CC needs to verify and match the identity information of the data provider before performing CKKS operations to assure the legitimacy of data provider’s identity. Subsequently, legitimate participants will undergo privacy calculations and the results will be returned to the requester of the privacy calculations.

The basic operations of homomorphic encryption include addition and multiplication, and the calculation process is as follows:

CKKS.Add$\left(c_{1},c_{2}\right)$. For $c_{1},c_{2}\in R_{q_{e}}^{2}$, Homomorphic addition of two ciphertext messages to obtain ciphertext $c_{\mathrm{add}}$:

$$c_{\mathrm{add}}\leftarrow c_{1}+c_{2}\;\left(\mathrm{mod}q_{\ell }\right).$$

${\mathrm{CKKS.Mult}}_{evk}\left(c_{1},c_{2}\right)$. The evaluation key $evk_{channel1}$ is required to perform the CKKS multiplication operation, we assume two ciphertext data $c_{1}$ and $c_{2}$. For $c_{1}=\left(b_{1},a_{1}\right)$, $c_{2}=\left(b_{2},a_{2}\right)\in R_{q_{e}}^{2}$, we can get $\left(d_{0},d_{1},d_{2}\right)=\left(b_{1}b_{2},a_{1}b_{2}+a_{2}b_{1},a_{1}a_{2}\right)\;\left(\mathrm{mod}\;q_{\ell }\right).$ We obtain the ciphertext $c_{\mathrm{mult}}$ after homomorphic multiplication:

$$c_{\mathrm{mult}}\leftarrow \left(d_{0},d_{1}\right)+\left\lfloor P^{-1}\cdot d_{2}\cdot evk_{channel1}\right\rceil \;\left(\mathrm{mod}\;q_{\ell }\right).$$

The specific content of multiplication ciphertext $c_{\mathrm{mult}}$ is as follows:

(14) $$\begin{array}{cc} c_{mult}= & \left(ct_{0},ct_{1}\right)\\ = & (d_{0}+\left\lfloor -P^{-1}\cdot d_{2}\cdot a^{\prime }\cdot s+P^{-1}\cdot d_{2}\cdot e^{\prime }+d_{2}\cdot s^{2}\right\rceil ,\\ \; & \;d_{1}+\left\lfloor P^{-1}\cdot d_{2}\cdot a^{\prime }\right\rceil ) \end{array}$$

Step 5: Decrypt encrypted data and decode it

The CKKS PN of the privacy computation requester from channel 1 decrypts and decodes the homomorphic encryption operation result.

The data decryption process is as follows:

${\mathrm{CKKS.Dec}}_{sk}\left(c\right)$. For c=(b,a), the CKKS PN of channel 1 decrypts ciphertext *c* with $sk_{channel1}$ to obtain polynomial plaintext information. The calculation process is shown in Equation (15).

(15) $$m=b+a\cdot s\;(\mathrm{mod}\;q_{\ell }).$$

The decoding process is as follows:

CKKS.Decode(m;Δ). For a given plaintext polynomial m(X)∈R, it is necessary to first use σ for canonical embedding to obtain σ(m), then remove the scaling factor with $\Delta^{-1}\cdot \sigma \left(m\right)$ and finally use π for projection mapping to obtain the message vector *z* as follows:

$$z\leftarrow \pi \left(\Delta^{-1}\cdot \sigma \left(m\right)\right)\in C^{N/2}$$

### 5.4. Access Permission Management

This section will introduce the specific process of managing access permissions for IoT devices. Managing access permissions for IoT devices includes IoT devices permission update and revocation. Our solution is based on DID to implement access control for IoT devices. The DID documents registered on the blockchain do not involve permission information. Our scheme achieves access permission management for IoT devices through the DID status list and attribute list managed by the AC.

#### 5.4.1. Permission Update

When the role or access scope of an IoT device needs to be changed, permission updates will be implemented through the following steps.

Step 1: Trigger permission update request

The IoT device submits a permission update request to the AC with $Request_{update}\left(DID,attribute^{\prime },sign\left(attribute^{\prime },timestamp\right)\right)$. The request information includes DID information, new permission attribute information $attribute^{\prime }$, and signature information with a timestamp $sign(attribute^{\prime },timestamp)$.

Step 2: Verification and execution

The AC verifies the validity of the request signature and the correctness of the new permission attributes $Verify_{update}\left(sign\left(attribute^{\prime },timestamp\right),attribute^{\prime }\right)$. After verification, the AC updates the attribute list information bound to the DID and generates an update event log.

Step 3: Issue new credentials

After the permissions are updated, the AC synchronizes the key operation hash of the permission update to the blockchain to achieve tamper-proof auditing. At the same time, the AC needs to issue verifiable credentials containing the new permissions to IoT devices to ensure that the devices can prove their latest permissions to resource providers.

#### 5.4.2. Permission Revocation

When the private key of an IoT device is leaked, the device is scrapped, or it needs to be permanently disabled, we need to revoke the permissions of the IoT device. This is achieved through the DID status list in the AC to revoke DID permissions. The permission revocation is implemented through the following steps.

Step 1: Initiate permission revocation

Permission revocation can be initiated autonomously by IoT devices or directly by AC $Request_{revocation}\left(DID,type_{revocation}\right)$. To facilitate subsequent traceability of permission revocation operations, permission revocation must provide the revocation type $type_{revocation}$ (e.g., private key leakage or device scrapping).

Step 2: Update DID status list

When updating the DID status list, the system first checks the current status of the target DID, then marks the target DID status as revoked in the DID status list and deletes the attribute list information bound to the target DID. At the same time, it records the revocation timestamp, administrator information who performed the revocation operation, revocation reason type, and other information. Additionally, the verifiable credentials associated with the DID must be added to the verifiable credential revocation list.

Step 3: Broadcast the results of permission revocation

To prevent IoT devices whose permissions have been revoked from continuing to exchange data with other devices, the AC will broadcast the permission revocation results, and IoT devices will terminate data exchange with related devices based on the revocation results.

Our scheme model involves the trusted registration of IoT devices, secure and efficient data sharing among IoT devices within the same domain, and privacy-protected data collaboration processes among IoT devices across domains. It meets the data sharing needs within and across IoT domains, providing a secure, efficient, and trusted data sharing solution for IoT devices.

## 6. Security Analysis

### 6.1. Privacy Protection

The system model of this scheme adopts the blockchain multi-channel mechanism to achieve the security of privacy computation between IoT devices in different channels by isolating different categories of IoT devices using blockchain channels so that they cannot directly interact with each other and uses the CKKS homomorphic encryption algorithm to make them carry out secure and private computation without exposing their own data so that our scheme is capable of achieving data privacy security in IoT devices during the data sharing process.

Furthermore, to enhance identity privacy protection, this scheme integrates DID into the IoT devices identity management process. Each IoT device autonomously generates its own DID and registers it with the blockchain through an authoritative center. This approach ensures that the IoT device takes total control of its own identity rather than having to rely on a centralized identity provider. Since DIDs are stored on the blockchain, they provide an anti-tamper and authenticatable identity mechanism, effectively preventing unauthorized identity manipulation and improving the security and privacy of IoT devices sharing.

### 6.2. IND-CPA Security

**Theorem** **1.** *Assuming that the RLWE problem holds, the system model proposed in this scheme is IND-CPA secure [31].*

**Proof.**  To demonstrate the IND-CPA security of our scheme, we begin by assuming the existence of an adversary *A* that is capable of compromising the IND-CPA security within polynomial time and with non-negligible advantage. Based on this assumption, we construct a simulator *B* that leverages *A*’s capabilities to solve the underlying RLWE problem.

Initialization: Select the polynomial ring $R=Z\left[x\right]/\left(x^{n}+1\right)$ and a scaling factor Δ. Let p>0 be a fixed base and $q_{0}$ be a modulus. Define a modulus chain $Q=q_{0}\cdot q_{1}\cdot \cdots \cdot q_{L}$, where $q_{\ell }=p^{\ell }\cdot q_{0}\;$ for 0<ℓ≤L.

Setup: The simulator *B* receives an RLWE instance (a,b), where b=a·s+e or *b* is uniformly random. *s* is a secret vector, and *e* is a small noise term. Set μ = 1 if b=a·s+e, and μ = 0 if *b* is a uniformly random value.

Query Phase 1: The adversary *A* can arbitrarily select a plaintext message *m* to submit to the oracle. The oracle will use the encryption key pk, which is known to the challenger but unknown to the adversary, to run the encryption algorithm and return the generated ciphertext ct to adversary *A*.

Challenge: The adversary *A* selects plaintexts $m_{0},m_{1}$ and submits them to *B*. *B* randomly selects r∈{0,1} and encrypts $m_{r}$ to obtain the following ciphertext:

(16) $$C^{*}=\left(c_{1},c_{2}\right)=\left(b\cdot v+m_{r}+e_{0},a\cdot v+e_{1}\right)$$

Query Phase 2: The adversary *A* can continue to send encrypted requests to the oracle.

Guess: The adversary *A* outputs $r^{\prime }$ as its guess for *r*. If the adversary *A* can successfully guess *r*, then B considers b=a·s+e, otherwise *B* considers *b* to be a uniformly random value.

Let μ=1 (i.e., b=a·s+e), then *A*’s success probability is

(17) $$Pr\left[r^{\prime }=r|\mu =1\right]=\frac{1}{2}+\epsilon$$

When μ=0 (i.e., *b* is uniformly random), the probability of *A* correctly guessing is

(18) $$Pr\left[r^{\prime }=r|\mu =0\right]=\frac{1}{2}$$

Thus, the advantage of simulator *B* is given by the following:

(19) $$\begin{array}{cc} Adv_{B}^{RLWE} & =\left|\frac{1}{2}Pr\left[B=1|\mu =1\right]+\frac{1}{2}Pr\left[B=0|\mu =0\right]\right|\\ \; & =\left|\frac{1}{2}Pr\left[r^{\prime }=r|\mu =1\right]+\frac{1}{2}Pr\left[r^{\prime }=r|\mu =0\right]-\frac{1}{2}\right|\\ \; & =\left|\frac{1}{2}\left(\frac{1}{2}+\epsilon \right)+\frac{1}{2}\left(\frac{1}{2}\right)-\frac{1}{2}\right|\\ \; & =\frac{\epsilon }{2} \end{array}$$

If *A* has a non-negligible advantage ϵ in breaking IND-CPA security, then *B* has a non-negligible advantage $\frac{\epsilon }{2}$ in solving the RLWE problem. This contradicts the RLWE assumption, proving that our scheme satisfies the IND-CPA security model. □

### 6.3. Cybersecurity Attack Analysis

The IoT trusted data sharing scheme based on multi-channel blockchain proposed in this paper effectively defends against cybersecurity attacks such as replay attacks, Sybil attacks, and distributed denial-of-service (DDoS) attacks through systematic design.

In terms of replay attack defense, the scheme relies on DID challenge–response authentication and timestamps to counter replay attacks. The challenge–response authentication method requires signing and verifying a random number during each authentication process to prevent replay attacks on identity verification. Additionally, the scheme uses timestamps to block duplicate or expired requests.

In terms of Sybil attack defense, the solution uses DID and blockchain channel mechanisms to verify and manage IoT devices. Each IoT device must register a unique on-chain anchored DID identity, and the DID registration process requires strict certification by the AC to ensure the legitimacy of the IoT device. Additionally, the blockchain multi-channel mechanism further strengthens defense. Devices from different domains are isolated into independent blockchain channels, and each channel implements strict node access control to defend against Sybil attacks.

In terms of DDoS attack defense, the solution employs a distributed architecture and blockchain channel isolation mechanism to safeguard against DDoS attacks that threaten system availability. The multi-channel mechanism naturally divides network, preventing system-wide paralysis caused by DDoS attacks. Data sharing within and across domains requires the participation of PNs. The blockchain contains a large number of distributed PNs, ensuring the system’s normal operation even if some PNs are attacked. The data storage layer uses the IPFS distributed file system, where original files are distributed across multiple nodes. Attackers cannot destroy stored data by attacking a single storage node, thereby defending against DDoS attacks.

### 6.4. Correctness Analysis

A CKKS encryption/decryption scheme’s correctness relies on controlling the error terms during encryption and ensuring the accuracy of the encoding/decoding processes. The detailed analysis is as follows:

Encryption Process: The ciphertext $c=\left(c_{0},c_{1}\right)=v\cdot pk+\left(m+e_{0},e_{1}\right)$, where pk=(b,a)=(−as+e,a). Expanding this yields the following:

$$\begin{array}{cc} c_{0} & =v\cdot b+m+e_{0}=v\left(-as+e\right)+m+e_{0},\\ c_{1} & =v\cdot a+e_{1}. \end{array}$$

Decryption Process: For the ciphertext data $c=(c_{0},c_{1})$, we decrypt it using the private key sk=(1,s), and the decryption process is as follows:

$$\begin{array}{cc} c_{0}+c_{1}\cdot s & =\left[v\left(-as+e\right)+m+e_{0}\right]+\left(va+e_{1}\right)s\\ & =-vas+ve+m+e_{0}+vas+e_{1}s\\ & =m+ve+e_{0}+e_{1}s\;\left(\mathrm{mod}\;q_{\ell }\right)\\ & \approx m \end{array}$$

Error-bound Discussion: An encryption *c* of *m* will satisfy $\langle c,sk\rangle =m+e\;\left(\mathrm{mod}\;q_{L}\right)$. Encryption noise *e* is bounded by $B_{\mathrm{begin}}=8\sqrt{2}\sigma N+6\sigma \sqrt{N}+16\sigma \sqrt{hN}$, where the constant $B_{begin}$ denotes an encryption bound. If $c\leftarrow {\mathrm{CKKS}.\mathrm{Enc}}_{pk}\left(m\right)$ and m←CKKS.Encode(z;Δ) for some $z\in Z{\left[i\right]}^{N/2}$ and $\Delta >N+2B_{\mathrm{begin}}$, then $\mathrm{CKKS}.\mathrm{Decode}({\mathrm{CKKS}.\mathrm{Dec}}_{sk}\left(c\right))=z.$ Let $\left(c_{i},\ell ,\nu_{i},B_{i}\right)$ be encryptions of $m_{i}\in S$ for i=1,2. For addition of $c_{1}$ and $c_{2}$, the error in the output ciphertext is limited to the sum of the two errors in the input ciphertext. For the multiplication of $c_{1}$ and $c_{2}$ with an error bounded by ${∥m_{1}e_{2}+m_{2}e_{1}+e_{1}e_{2}+e^{\prime \prime }∥}_{\infty }^{\mathrm{can}}+B_{\mathrm{scale}}\leq \nu_{1}B_{2}+\nu_{2}B_{1}+B_{1}B_{2}+P^{-1}\cdot q_{\ell }\cdot B_{\mathrm{ks}}+B_{\mathrm{scale}}$, where $B_{ks}=8\sigma N/\sqrt{3}$ and $B_{scale}=\sqrt{N/3}\cdot \left(3+8\sqrt{h}\right)$.

## 7. Performance and Evaluation

### 7.1. Functionality Comparison

A comparison of this scheme with existing schemes is shown in Table 4. Our proposed scheme in this paper achieves multidimensional innovation in the core requirements of trusted data sharing in IoT. Below is a detailed comparison from the perspective of functional architecture:

#### 7.1.1. Autonomous Identity Control

Compared to scheme [18], scheme [22], and scheme [32], this paper adopts DID technology to replace the traditional identity management approach, thus strengthening the system’s security and privacy protection. Traditional identity management relies on a centralized authentication authority, which carries the danger of a single-site failure and may lead to the disclosure of private user identity information during data sharing. DID technology, on the other hand, stores identity information through the blockchain, allowing users to independently take control of their identity information rather than depending on a third-party organization to store it, which enhances the decentralization of identity management and reduces the risk of identity forgery and tampering.

#### 7.1.2. Blockchain Storage Capacity Limit

Scheme [18], scheme [22], and scheme [32] all utilize the cloud to store data and upload the index information to the blockchain, although this scheme solves the blockchain storage capacity limitation problem, but storing data through the cloud has the dangers of single point of failure.

This paper innovatively constructs a hybrid architecture of on-chain deposit and off-chain storage. Original IoT devices data encrypted and saved in IPFS network, unique data fingerprints are generated through content addressing, and only key data information is stored on the chain, which significantly improves the storage capacity of the system. When accessing the data, the data requester needs to verify the requester’s authority through a smart contract and can only access the shared data on IPFS after passing the verification. This hybrid architecture ensures trustworthiness while dramatically reducing storage costs compared to pure on-chain data storage solutions, providing a viable path for massive data storage in large-scale IoT devices.

#### 7.1.3. Cross-Domain Data Sharing Scenario

Existing schemes such as scheme [18], scheme [22], and scheme [32] do not fulfill the need for sharing data in cross-domain scenarios. In this paper, we propose a cross-domain sharing framework based on channel segregation and CKKS encryption. For cross-domain data sharing, cross-domain data sharing is realized through PN. Using CKKS to homomorphic encrypt cross-domain shared data reduces the computational pressure on both sides of the data by implementing homomorphic encryption operations in a third-party computing center, while CKKS enables data to be homomorphically computed in the form of ciphertexts in a third-party computing center to avoid data privacy leakage.

### 7.2. Computation Cost Comparison

In this subsection, the computational overhead of different schemes will be compared, and the meaning of symbols related to computational overhead is shown in Table 5. Table 6 shows the computation overhead of our scheme compared to other schemes, including the computation overhead comparison of key generation, data encryption and data decryption.

From the computation overhead comparison results, we can find that our scheme performs well in computation overhead compared to other schemes while realizing the trusted data sharing of IoT devices. The main computation overhead of this scheme is to utilize CKKS to realize the data sharing between cross-domain IoT devices, so this scheme has good feasibility in terms of computation overhead.

### 7.3. Experimental Analysis

This experiment uses the tenseal library to implement performance testing of CKKS. The environment of the experiment includes an Intel (R) Core (TM) i7-12800HX 2.00 GHz CPU, 16 GB RAM, Pycharm software environment, Ubuntu 20.04, hyperledge fabric 2.2 and Python interpreter version 3.12. The configuration information of the virtual machine includes 2 GB of memory, a single-core processor, and 20 GB of disk space. In addition, IPFS is utilized for distributed data storage.

This testing experiment includes testing the CKKS key generation time under different cases, testing the CKKS encryption and decryption time under different cases, and testing the relationship between ciphertext size and plaintext size under different cases. The parameter configurations for different cases are shown in the Table 7. Performance tests are also conducted on AES encryption and decryption efficiency and IPFS file upload and download efficiency.

**Figure 4:**
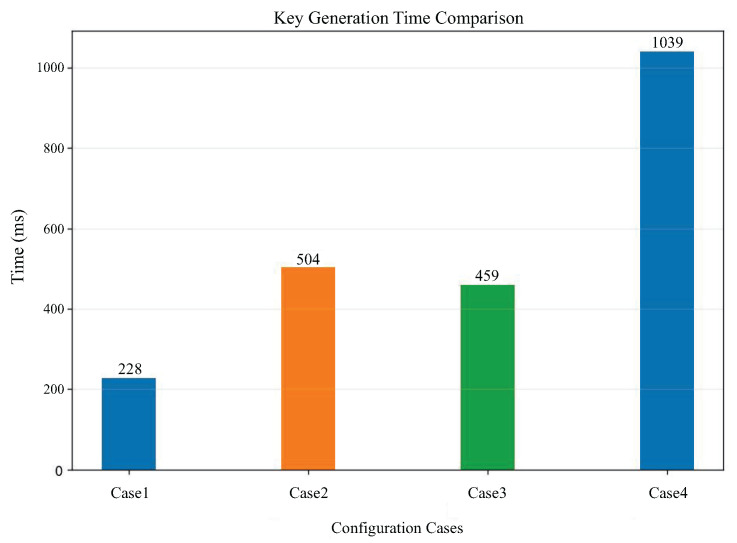
Comparison of key generation time under different cases.

The CKKS key generation time is presented in Figure 4. It can be observed that the increase in the polynomial modulus directly affects the key generation time. The larger the polynomial modulus, the longer the key generation time. From the comparison between case 2 and case 3, we can see that the difference in coefficient modulus also affects the key generation time.

**Figure 5:**
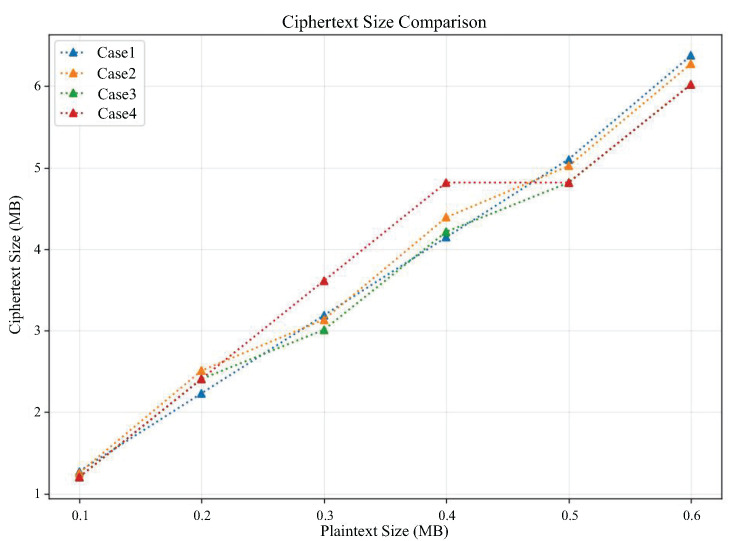
The relationship between ciphertext size and plaintext size in different cases.

From Figure 5, it can be seen that the ciphertext size and plaintext size of all cases are linearly correlated, and ciphertext size grows as the plaintext size grows. Therefore, this linear relationship makes the size of ciphertext data after encryption of plaintext data acceptable.

**Figure 6:**
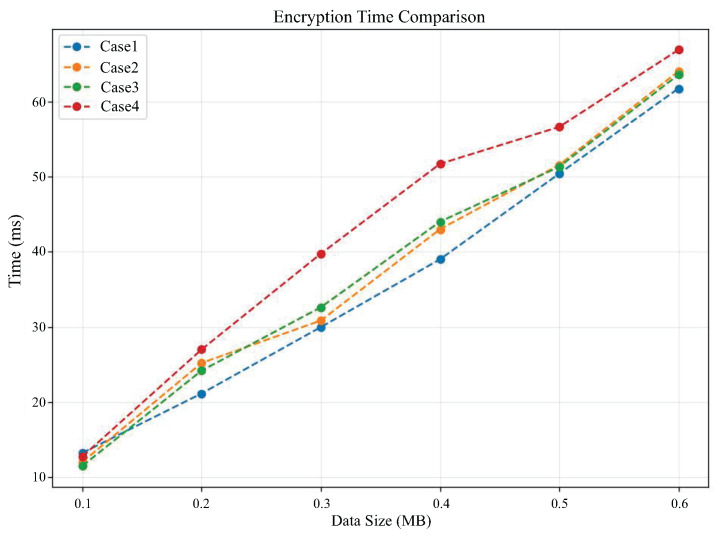
Comparison of encryption time under different cases.

**Figure 7:**
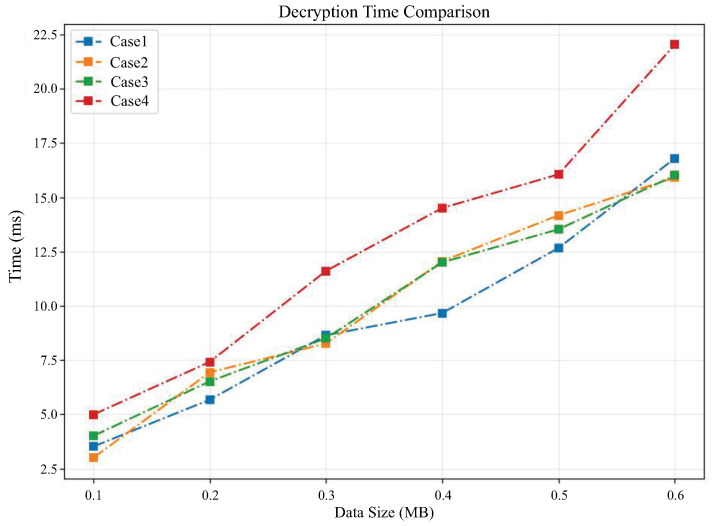
Comparison of decryption time under different cases.

Figure 6 and Figure 7 compare the encryption time and decryption time, respectively, as the data size changes under different cases. From Figure 6, it can be seen that the encryption time in all cases increases with the amount of data. It can be observed that case 4 requires significantly more encryption time than other cases, which reflects that the encryption time is also related to the value of polynomial modulus. The higher the value of polynomial modulus, the longer the encryption time will become. From Figure 7, it can also be observed that the decryption time required for case 4 is generally higher than the other three cases.

**Figure 8:**
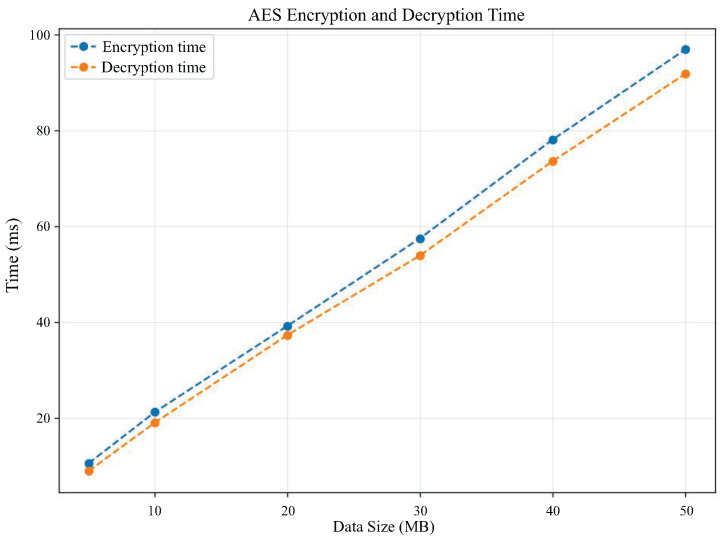
Trend graph of AES encryption and decryption times with file size.

AES symmetric encryption of the original data file is required before uploading the data to IPFS. As shown in Figure 8 is the trend graph of AES symmetric encryption and decryption time with the change of file size, from the experimental results can be found that the encryption and decryption time and file size are linearly correlated, and the encryption and decryption time is basically consistent.

**Figure 9:**
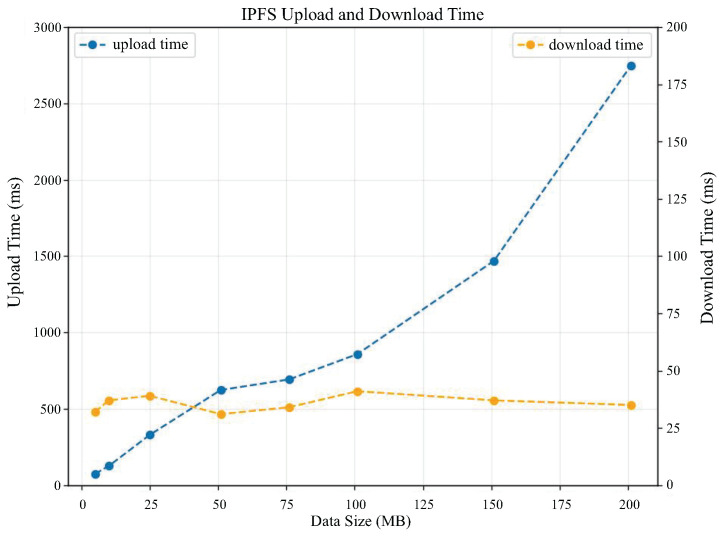
Trend graph of IPFS file upload and download times with file size.

To conduct performance tests for file uploads and downloads on IPFS, we set up a private network with three IPFS peers on a local virtual machine. These three IPFS peers are connected via a swarm key. We then conducted performance testing by uploading and downloading eight data files of different sizes to and from IPFS (file sizes were 5 MB, 10 MB, 25 MB, 50 MB, 75 MB, 100 MB, 150 MB, and 200 MB). The performance test results for IPFS file upload and download efficiency are shown in Figure 9. From the figure, we can see that the time required for uploading IPFS files is linearly related to the file size, and the download time of the file basically stays around 35 ms, the experimental results can reflect that this program is feasible to utilize IPFS for data storage.

The environment configuration required for Hyperledger Fabric 2.2 in this experiment includes Docker 28.0.1 and Docker Compose 1.29.1 configuration information. The Raft consensus mechanism is used. The fabric network is configured with three channels and two organizations, each organization containing four peers. Organization 1 is joined to Channel 1 (for internal business), Organization 2 is joined to Channel 2 (for internal business), and both Organization 1 and Organization 2 are joined to Channel 3 (for general business). Channels are managed based on the business requirements of different organizations. Additionally, we tested the fabric network, with the test results showing a throughput of 630.6 tps. This test results demonstrate the feasibility of hyperledger fabric for IoT data sharing.

Overall, the above experiments reflect that different parameter configurations directly affect the generation of keys and the encryption and decryption time of data. While the CKKS encryption algorithm becomes more secure as the polynomial modulus grows, the efficiency of the overall algorithm also decreases significantly as the polynomial modulus value grows. Therefore, suitable parameter configurations can be selected to balance security and efficiency requirements in different scenarios. Using IPFS to realize secure sharing of big data files can effectively solve the blockchain capacity limitation problem. In addition, the test results of the hyperledger fabric blockchain platform used in our scheme further validate the feasibility of this scheme for data sharing in IoT.

## 8. Conclusions

Aiming at the different data sharing needs within and across domains in the current IoT, this paper proposes a trusted data sharing scheme for IoT based on multi-channel blockchain to solve the dual challenges of efficient intra-domain collaboration and cross-domain privacy protection in IoT. The scheme achieves data isolation for IoT devices in different domains by introducing a multi-channel ledger isolation mechanism. Intra-domain IoT devices support efficient data sharing and secure access control through hybrid encryption and automatic execution of smart contracts. The cross-domain IoT devices realize privacy computation in ciphertext state and secure data sharing through CC with the help of CKKS fully homomorphic encryption algorithm.

In addition, this solution combines DID technology to empower IoT devices with autonomous identity management capabilities, avoiding the danger of centralized identity servers creating a single point of privacy disclosure. Meanwhile, through the co-storage mechanism of IPFS and blockchain on-chain and off-chain, it addresses the limited storage capability of blockchain and enables the efficient and trustworthy sharing of big data files. Security analysis shows that our scheme satisfies the IND-CPA security model. Future work will focus on optimizing the computational efficiency of the CKKS algorithm to support more complex cross-domain collaboration scenarios and exploring a lightweight verification mechanism based on zero-knowledge proof.

## Figures and Tables

**Table 1:** Threat model adversary capability.

Adversary	Capabilities	Security Goals Not Breached
Outsider	Conduct DDoS and other attacks to disrupt network availability and eavesdrop on public communication channels.	Data confidentiality remains protected, and authentication mechanisms prevent unauthorized system access.
Compromised PN	Attempt to tamper with the CKKS encryption process for shared data.	Computation integrity preserved through blockchain consensus validation.
Compromised CC	Attempt cryptanalytic attacks against CKKS ciphertexts to recover plaintext data.	The data transmitted to CC is encrypted using the CKKS key, and CC cannot solve the RLWE hard problem.
Colluding PN + CC	CC and PN jointly forge and tamper with CKKS encryption operation data in CC.	The immutability of blockchain enables traceability and auditing of data operations.

**Table 2:** Comparison between single-channel and multi-channel blockchain architectures.

Evaluation Dimension	Single-Channel Architecture	Multi-Channel Architecture
Transaction Delays	All transactions are sorted globally, and high competition leads to high delay fluctuations.	Different channels do not affect each other, and channel transactions are processed in parallel.
Scalability	Limited scalability due to global state replication and single consensus group.	New domains added as independent channels without global impact.
Consensus Efficiency	Single consensus group processes all transactions.	Configure different consensus mechanisms for each channel according to business needs.
Resource Utilization	Need to store global transaction ledger data.	Only store data for channels they participate in.
Security Isolation	Rely on policy-based access control to isolate data.	Each channel is an independent ledger with natural isolation.
Fault Containment	A catastrophic failure would affect all data sharing operations.	Issues contained within affected channel, other channels unaffected.
Cross-Domain Collaboration	A single channel is insufficient to achieve secure and efficient cross-domain collaboration.	Achieve cross-domain collaboration through multi-channel management.

**Table 3:** Related symbol definitions.

Symbol	Description
*N*	Polynomial ring dimension
*Q*	Modulus chain
Δ	Scaling factor
*a*	Public polynomial
pk	Public key for data encryption
rlk	Relinearization key
*m*	Plaintext polynomial data
$\left(c_{0},c_{1}\right)$	Ciphertext pair
σ	Canonical embedding
π	Mapping function
*v*	Random polynomial generated during encryption
*e*	Noise term
R	Polynomial ring $Z\left[X\right]/\left(X^{N}+1\right)$
$R_{Q}$	Modular polynomial ring $Z_{Q}\left[X\right]/\left(X^{N}+1\right)$
*L*	Number of modulus chain levels

**Table 4:** Functionality comparison with existing data sharing schemes.

Scheme	Decentralized Identity	Platform	Cross Domain Sharing	Privacy Protection
Scheme [18]	–	Hyperledger fabric	–	Paillier
Scheme [22]	–	Blockchain	–	CP-ABE
Scheme [32]	–	Federated Blockchain	–	ABE
Proposed Scheme	✔	IPFS, Hyperledger fabric	✔	CKKS

**Table 5:** Computation overhead symbol meaning.

Symbol	Description
*n*	The number of attributes
$T_{H}$	Hash operation time
$T_{\exp}$	Exponentiation operation time
$T_{P}$	Pairing operation time
$T_{m}$	Scalar multiplication operation time
$T_{d}$	Scalar division operation time
$T_{s}$	Scalar subtraction operation time
$T_{a}$	Scalar addition operation time
$T_{M}$	Multiplication operation time
$T_{D}$	Division operation time
$T_{A}$	Addition operation time
$T_{\mathrm{map}}$	Mapping operation time

**Table 6:** Comparison of computational cost.

Scheme	Keygen	Encryption	Decryption
Scheme [18]	$2T_{m}+2T_{s}$	$2T_{\exp}+T_{M}+T_{a}$	$T_{\exp}+T_{M}+T_{D}+T_{S}$
Scheme [22]	$\left(2n+3\right)T_{\exp}+T_{P}+nT_{H}+nT_{M}$	$nT_{H}+\left(2n+2\right)T_{\exp}$	$2T_{P}$
Scheme [32]	$4T_{\exp}+3T_{d}+T_{s}+T_{a}$	$T_{\exp}+T_{P}+T_{m}$	$T_{P}+T_{M}+T_{D}$
Proposed Scheme	$3T_{\mathrm{mul}}+3T_{A}+T_{\exp}$	$2T_{M}+2T_{A}+2T_{\mathrm{map}}$	2$T_{M}+T_{A}+2T_{\mathrm{map}}$

**Table 7:** Parameter configurations for CKKS homomorphic encryption.

Name	Poly Modulus Degree	Coefficient Modulus	Scale
Case1	8192	[60, 40, 40, 60]	$2^{40}$
Case2	16,384	[60, 40, 40, 60]	$2^{40}$
Case3	16,384	[50, 40, 40, 50]	$2^{40}$
Case4	32,768	[50, 40, 40, 50]	$2^{40}$

## Data Availability

The original contributions presented in the study are included in the article, and further inquiries can be directed to the corresponding author.
